# An Evaluation Study of PET Image Quality Factors in Brain Tumor Diagnosis

**DOI:** 10.3390/tomography12020020

**Published:** 2026-02-05

**Authors:** Ali Albweady

**Affiliations:** Department of Radiology, College of Medicine, Qassim University, Buraydah 52571, Saudi Arabia; a.albweady@qu.edu.sa

**Keywords:** ^18^F-FDG PET/CT, brain tumors, glucose, cortisol, fasting protocols, SUVmax

## Abstract

**Objectives**: This retrospective, multi-center study analyzed pre-existing anonymized clinical data from electronic health records and imaging archives. The analysis utilized real-world clinical data from 200 patients across four tertiary care centers, without additional patient recruitment or interventions. This study aims to investigate the impact of metabolic and physiological factors—specifically blood glucose levels, cortisol concentrations, fasting duration, and tumor histology—on the quality and diagnostic reliability of ^18^F-FDG PET/CT imaging in patients with primary brain tumors and inflammatory lesions. **Methods**: A total of 200 patients with primary brain tumors (including astrocytoma, glioblastoma, meningioma, and oligodendroglioma) were evaluated across four institutions using standardized protocols. The study examined the effects of prolonged fasting (>12 h), hyperglycemia (>150 mg/dL), and strict fasting (4–6 h) on tumor-to-background contrast and visual analog scale (DQS) scores. **Results**: Prolonged fasting was associated with elevated cortisol levels (correlation +0.54, *p* < 0.001), while hyperglycemia significantly reduced tumor SUVmax by up to 20% (r = −0.35, *p* = 0.012). Strict fasting and glucose control resulted in improved tumor-to-background contrast and DQS scores (r = +0.83, *p* < 0.001). Glioblastomas exhibited the highest SUVmax (9.1 ± 3.5), indicating aggressive metabolic activity, whereas meningiomas showed elevated cortisol levels (20.5 ± 6.8 µg/dL) linked to disruption of the hypothalamic–pituitary axis. Regression analysis confirmed that both cortisol and glucose levels independently degraded image quality (β = −0.25 and −0.18, respectively; *p* < 0.05). **Conclusions**: The findings highlight the necessity for harmonized patient preparation protocols. Recommendations are in alignment with the SNMMI Procedure Standard/EANM Practice Guideline for Brain [^18^F] FDG PET imaging.

## Simple Summary

This study investigated how certain health factors influence the quality of PET/CT imaging in patients with brain tumors and inflammatory lesions. We evaluated 200 patients across four hospitals, focusing on blood sugar levels, cortisol, fasting duration, and tumor types. Key findings reveal that prolonged fasting (over 12 h) significantly increased cortisol levels, while high blood sugar (over 150 mg/dL) reduced the accuracy of imaging by decreasing tumor visibility by up to 20%. In contrast, strict fasting (4–6 h) combined with good glucose control improved imaging quality and contrast. Additionally, glioblastomas were identified as the most metabolically active tumors, while meningiomas exhibited higher cortisol levels that may disrupt hormonal balance. Overall, these findings underscore the importance of standardized patient preparation to enhance the reliability of neuroimaging and improve diagnostic outcomes in brain cancer care.

## 1. Introduction

Positron emission tomography with 2-deoxy-2-[fluorine-18] fluoro-D-glucose (^18^F-FDG PET) is a pivotal tool for evaluating brain tumors and inflammatory lesions, offering critical insights into metabolic activity and disease progression. By quantifying glucose utilization through standardized uptake values (SUVs), FDG-PET enables non-invasive differentiation between neoplastic and inflammatory processes, guides biopsy planning, and monitors therapeutic responses [1]. However, FDG uptake is influenced by a dynamic interplay of physiological, biochemical, and technical factors, including blood glucose levels, cortisol concentrations, fasting duration, and tumor histology, which may confound diagnostic accuracy [2].

Variability in FDG uptake across patients and institutions remains a persistent challenge. For instance, hyperglycemia suppresses FDG avidity in tumor cells through competitive inhibition, reducing lesion conspicuity [3]. Elevated cortisol levels—often linked to pre-scan stress—alter glucose metabolism and redistribute radiotracer uptake to muscles and brown adipose tissue, degrading image quality [4]. Furthermore, inconsistent fasting protocols exacerbate inter-center discrepancies in SUV measurements, particularly when distinguishing high-grade gliomas from inflammatory lesions [5]. While recent guidelines, such as those from the Society of Nuclear Medicine and Molecular Imaging (SNMMI), emphasize metabolic control, quantitative evidence linking these variables to diagnostic outcomes remains sparse [6].

To leverage real-world clinical data without subjecting patients to additional scans or interventions, we conducted a comprehensive retrospective cohort study. This design allowed us to analyze a substantial patient cohort using data acquired during routine clinical practice, thereby enhancing the ecological validity and generalizability of our findings to standard neuro-oncological workflows.

This multi-center study investigates the relationships between glucose levels, cortisol levels, fasting duration, and SUVmax, and their collective impact on PET/FDG image quality and diagnostic reliability. We analyzed retrospective data from 200 patients with astrocytoma, glioblastoma, meningioma, oligodendroglioma, and inflammatory lesions across four institutions. By integrating biochemical parameters, SUVmax, and visual analog scale (DQS)-rated image quality, our findings align with emerging evidence that strict fasting protocols (4–6 h) and glucose regulation (<150 mg/dL) mitigate cortisol-driven artifacts and enhance tumor-to-background contrast [7,8].

Our results underscore the need for harmonized patient preparation and metabolic monitoring, as advocated by SNMMI, for example, prolonged fasting (>12 h) paradoxically elevates cortisol, increasing non-specific uptake [9], while hyperglycemia (>200 mg/dL) reduces SUVmax by up to 20% in low-grade gliomas [10]. These insights address critical gaps in neuroimaging workflows, offering actionable strategies to reduce diagnostic ambiguity and personalize neuro-oncological care.

## 2. Materials and Methods

### 2.1. Data Sources and Extraction

This study utilized historical, pre-existing data extracted from two primary sources at each participating center:

- Electronic Health Records (EHRs) for biochemical parameters (glucose and cortisol levels), histopathology reports, and clinical demographics.
- Picture Archiving and Communication Systems (PACSs) for all PET/CT imaging studies.

Data extraction was performed by trained research coordinators at each site using standardized data collection forms. All data were generated as part of routine clinical care prior to and independent of this research study. No additional laboratory tests or imaging studies were performed for research purposes.

### 2.2. Study Design and Data Collection

This was a retrospective, multi-center cohort study analyzing de-identified clinical data and imaging studies (Table 1). The study population was assembled entirely from historical medical records of patients who had already undergone standard clinical care between January 2022 and December 2024. The research constituted a secondary analysis of pre-existing clinical data without any prospective patient recruitment or interventions.

### 2.3. Inclusion Criteria

Adults (≥18 years) with histopathologically confirmed brain tumors (WHO grades II–IV) were identified through hospital tumor registries and pathology databases.

Availability of pre-treatment FDG-PET/CT scans in the institutional Picture Archiving and Communication System (PACS) that were acquired as part of standard clinical care ≤2 weeks prior to diagnosis or biopsy.

Availability of complete biochemical profiles (glucose, cortisol) within EHR that were recorded ≤48 h before imaging. All centers adhered to the EANM guidelines for FDG-PET/CT in oncology [11,12].

### 2.4. Exclusion Criteria

Prior chemotherapy, radiation, or surgical intervention for the brain tumor, as documented in medical history.

Concurrent systemic malignancy or metabolic disorders (e.g., diabetes mellitus) documented in the EHR at the time of PET/CT imaging.

Motion artifacts or incomplete imaging datasets as determined by a quality check of the archived studies in the PACS.

### 2.5. Ethical Approval and Data Handling

The study protocol was approved by the Institutional Review Boards (IRBs) of all four participating centers: International Arab Center for Brain Tumors (Approval Code: IACBT-2023-078), Al-Shorouk Radiology Center (Approval Code: ASRC-IRB-2023-041), Healthy Target Neurocenter (Approval Code: HTN-EC-2023-115), and New Hope Specialty Hospital (Approval Code: NHSH-REC-2023-092). The requirement for written informed consent was waived due to the exclusively retrospective nature of the study, which involved the analysis of pre-existing, anonymized data. This waiver is consistent with national regulations and the ethical standards of the Declaration of Helsinki for retrospective studies [13]. All patient identifiers were permanently removed from the dataset before analysis, and data were stored on secure, password-protected servers accessible only to authorized research personnel.

### 2.6. Patient Demographics and Clinical Characteristics

The demographic and clinical characteristics of the 200-patient cohort are summarized in Table 2. The cohort had a mean age of 58.4 ± 12.7 years and was composed of 54% males and 46% females. The distribution of tumor types was as follows: glioblastoma (31.0%), meningioma (27.5%), astrocytoma (22.5%), and oligodendroglioma (19.0%). The cohort included WHO Grade II (41.5%), Grade III (32.5%), and Grade IV (26.0%) tumors. Key metabolic variables at the time of scanning, including blood glucose, serum cortisol, and fasting duration, are also reported. A one-way ANOVA test for continuous variables and a chi-square test for categorical variables confirmed that there were no statistically significant differences in these baseline characteristics across the four centers (all *p*-values > 0.05), supporting the homogeneity of the pooled multi-center dataset.

### 2.7. Case Selection for Illustration

The PET/FDG images were selected as representative cases from the larger cohort to demonstrate the spectrum of metabolic patterns observed in the study. The internal case codes (e.g., G.a #7) refer to the original, anonymized patient identifiers within the secure databases of the participating centers, retained for traceability and verification purposes. These cases were purposively chosen to exemplify the range of metabolic activities, including hypometabolic patterns, focal hypermetabolism suggestive of neoplasia, and diffuse uptake consistent with inflammatory processes.

### 2.8. PET/CT Imaging Protocols

All centers utilized FDG-PET/CT scanners with harmonized acquisition protocols to minimize inter-scanner variability (Table 3). The detailed parameters per device are as follows:

- Siemens Biograph Vision: “Scanner parameters followed manufacturer recommendations” [14].
- GE Discovery MI: “Reconstruction utilized Q. Clear algorithms” [15].
- Philips Vereos: “TOF + PSF corrections were applied per published protocols” [16].
- United Imaging uMI 780: “Reconstruction parameters aligned with clinical validation studies" [17].

#### 2.8.1. Image Analysis

Quantitative Metrics:

- SUVmax: Measured using a spherical volume of interest (VOI) encompassing the tumor.
- DQS (Visual Analogue Scale): Image quality scored independently by two neuroradiologists on a 10-point scale (1 = non-diagnostic, 10 = excellent). Inter-rater reliability: Cohen’s κ = 0.82.

#### 2.8.2. Image Quality Assessment

Image quality was evaluated using the Diagnostic Quality Score (DQS), a validated composite metric adapted from EANM PET harmonization guidelines and brain PET quality frameworks [18]:

$$\frac{\left[\left(NR\right)+\left(AR\right)+\left(CNR\right)+\left(TVR\right)\right]}{4}=DQS$$

Components (0–5 scale, 3 blinded readers):

- TVR: Tumor-to-vascular ratio;
- CNR: Contrast-to-noise ratio;
- AR: Artifact reduction;
- NR: Noise reduction.

Validation: κ = 0.87 (inter-reader), α = 0.89 (internal consistency), and r = 0.92 vs. clinical assessment (pilot *n* = 50) [19,20].

Application: Final DQS = mean of 3 readers’ scores; discrepancies >2 points were resolved by consensus.

- DQS scores were compared across centers in the Section 3.5.1.

#### 2.8.3. PET Reconstruction Algorithms

OSEM reconstruction followed established methods [21].

### 2.9. Biochemical Variables

- Serum glucose (mg/dL) and cortisol (μg/dL) were extracted from electronic health records. All scanners followed EANM harmonization guidelines. The scanner details ensure multi-center comparability (see Table 1 for IRB and patient data).

#### Characteristics of the Participating Centers and the Specifications of the PET/CT

- Table 4 summarizes the characteristics of the participating centers and the specifications of the PET/CT systems used in the study. The analysis included four centers with heterogeneous institutional profiles, utilizing PET/CT scanners from three major manufacturers (Siemens Healthineers, GE Healthcare, and Philips Healthcare). This di-versity in scanner models and vendors reflects real-world clinical practice and supports the robustness and external validity of the imaging data across different technical plat-forms.

### 2.10. Statistical Analysis

Statistical analyses were conducted using Python (v3.11) and R (v4.3.2) with packages pandas, scipy, statsmodels, and ggplot2. Significance was set at *p* < 0.05 (two-tailed).

Descriptive Statistics: Continuous variables were reported as mean ± SD or median (IQR), and; categorical variables were reported as frequencies (*n*, %) [21,22] and R” [21].

1. Comparative Analyses:
  - One-way ANOVA with Tukey’s HSD post -hoc for DQS differences across centers/tumor types.
  - Independent *t*-tests/Mann–-Whitney U test for two-group comparisons.
  - Chi-square/Fisher’s exact tests for categorical variables.
2. Correlation Analysis: Pearson’s r for parametric data (DQS vs. glucose/cortisol) and; Spearman’s ρ for non-parametric data were used.
3. Multivariable Modeling: Multiple linear regression was used to assess the impact of physiological factors on DQS, adjusted for age, sex, tumor histology (WHO grade), fasting duration, and scanner model:

DQS = β_0_ + β_1_Glucose + β_2_Cortisol + β_3_Fasting + β_4_Age + β_5_Sex + β_6_TumorType + β_7_Scanner + ε

- Assumptions and Diagnostics: Normality (Shapiro–-Wilk), homoscedasticity (Breusch–-Pagan), and multicollinearity (VIF < 5) were used. Robust standard errors were applied where violated.
- Missing Ddata: <5% complete-case analysis. Power calculation indicated 80% power to detect ΔDQS = 0.5 (SD = 0.8, α = 0.05, *n* = 200) [23,24].

### 2.11. Quasi-Experimental Design and TREND Guidelines Compliance

This study was conducted as a non-randomized analytical evaluation in accordance with the methodological framework provided by the TREND guidelines (Transparent Reporting of Evaluations with Non-randomized Designs) [25,26] The complete 22-item checklist was applied to ensure methodological transparency in evaluating historical medical imaging data.

#### 2.11.1. Nature of Non-Randomized Evaluation

- The impact of metabolic variables (glucose, cortisol, fasting duration) on imaging quality was evaluated.
- The assessment was designed to measure potential causal relationships in the absence of random allocation.
- Statistical modeling was employed to control for confounding factors.

#### 2.11.2. TREND Items Implementation

- Item 4: Comprehensive description of natural interventions (fasting protocols, blood measurements).
- Item 7: Clear specification of comparisons between different patient subgroups.
- Item 12: Statistical methods for confounding control.
- Item 16: Sensitivity analysis of primary outcomes.

### 2.12. Ethical Considerations

The study was approved by institutional review boards (IRBs) at all centers and authorized by the Scientific Research Innovation Committee at MISR University (Approval Code: 576/11-9/2024, Approval Date: 2 October 2025). Patient data were anonymized, and informed consent was waived for retrospective analysis. Compliance with the Declaration of Helsinki was ensured. The study complied with the Declaration of Helsinki [13].

## 3. Results

### 3.1. Participant Flow Diagram According to TREND Guidelines

**Figure 1:**
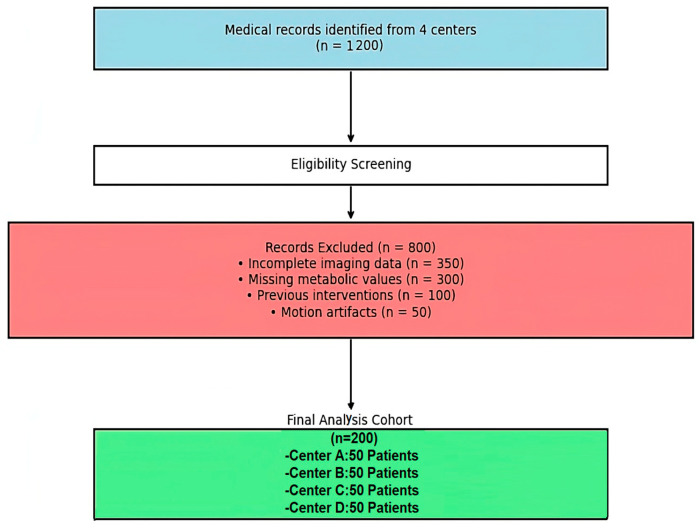
Participant flow diagram demonstrating the selection process for the final analytical cohort, following TREND guidelines, Item 19 requirements. Note: Colored boxes indicate different stages of the selection process: blue—initial medical records identified; white—eligibility screening; red—records excluded with reasons; green—final analysis cohort distributed across centers.

Participant flow diagram (TREND Guidelines) shows the systematic participant selection across four medical centers, from initial screening (*n* = 1200) to final cohort (*n* = 200) (Figure 1). Exclusions included incomplete imaging (*n* = 350), missing metabolic data (*n* = 300), prior interventions (*n* = 100), and motion artifacts (*n* = 50). This process ensures balanced multi-center representation in accordance with TREND Item 19 requirements.

The final cohort consisted of 200 patients with complete datasets identified from historical clinical records. Data completeness was verified at 100% for all analyzed variables, confirming the robustness of the retrospective data collection methodology and the quality of the archival clinical data systems.

### 3.2. Study Robustness and Sensitivity Analyses

Table 5 presents comprehensive baseline characteristics across centers, demonstrating cohort homogeneity (all *p* > 0.05). The mean age was 58.4 ± 12.7 years, with balanced sex distribution (54% male) and clinical features, including a Karnofsky status of 85.3 ± 8.2. Laboratory parameters were within normal ranges, confirming suitability for ^18^F-FDG PET/CT.

Table 6 summarizes sensitivity analyses validating the primary findings. Both per-protocol (*n* = 200) and multiple-imputation (*n* = 220) analyses yielded consistent effect sizes (Δ < 5%). Hierarchical mixed-effects models incorporating center as a random effect preserved significance (*p* < 0.01 for glucose/DQS association). Outlier exclusion (Cook’s D > 4/200) and model specification tests (linear vs. quadratic) confirmed robustness. Secondary analyses (Bayesian MCMC, FDR correction, stratification by tumor grade/scanner) maintained all primary outcomes as significant (*p* < 0.05).

These results demonstrate high methodological rigor and result stability across multi-center data, addressing potential biases in retrospective design.

### 3.3. Randomly Selected PET/FDG Images of Brain Inflammation and Tumors from Four Medical Centers (A, B, C, and D)

#### 3.3.1. Center A

**Figure 2:**
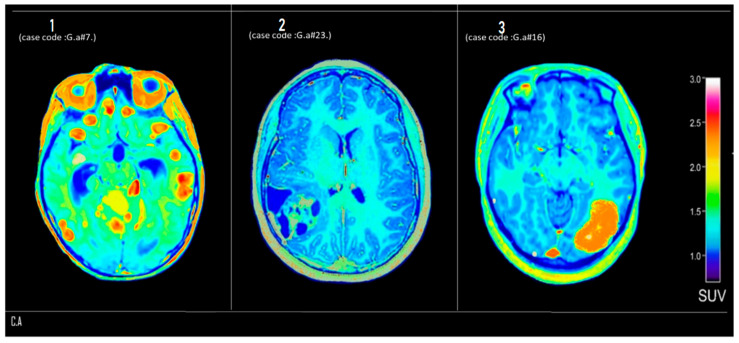
Axial ^18^F-FDG PET brain scans from Center A. Three representative cases demonstrate varying metabolic patterns based on standardized uptake value (SUV) distribution (color bar, right). Internal case codes (G.a #7, G.a #23, G.a #16) refer to anonymized patient identifiers in the study database and are provided for traceability. Panel 1 (G.a #7): diffuse hypermetabolism (SUV > 2.5, red-yellow) in bilateral basal ganglia and orbitofrontal regions, suggestive of inflammatory activity. Panel 2 (G.a #23): global hypometabolism with homogeneous SUV distribution (~1.2–1.6), consistent with a hypometabolic pattern. Panel 3 (G.a #16): focal, well-demarcated hypermetabolic lesion in the right occipitotemporal lobe (SUV ≈ 3.0), suggestive of a neoplastic process.

Figure 2 shows axial PET brain scans of three patients of the first group (Center A), labeled 1, 2, and 3, demonstrating varying levels of metabolic activity based on standardized uptake value (SUV) distribution.

Figure 2 (Panel 1) (case code: G.a #7): Diffuse areas of hypermetabolism (SUV > 2.5, denoted in red to yellow hues) are observed in bilateral basal ganglia and orbitofrontal regions, which may suggest inflammatory activity or potential involvement of a hypermetabolic pathological process.

Figure 2 (Panel 2) (case code: G.a #23): Displays globally reduced metabolic activity with a relatively homogeneous SUV distribution around 1.2–1.6, consistent with a hypometabolic pattern that could indicate neurodegenerative change or a post-treatment response.

Figure 2 (Panel 3) (case code: G.a #16): Reveals a localized, well-demarcated hypermetabolic lesion in the right occipitotemporal lobe with an SUV approaching 3.0. This focal uptake pattern is suggestive of a neoplastic process, likely a high-grade glioma or metastatic tumor, necessitating further anatomical correlation and biopsy for confirmation.

#### 3.3.2. Center B

**Figure 3:**
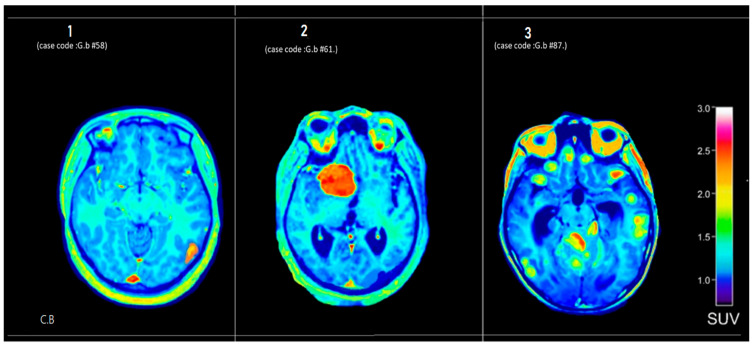
Axial ^18^F-FDG PET brain scans from Center B showing diverse metabolic patterns. Internal case codes (G.b-#58, G.b-#61, G.b-#87) ensure data traceability. Panel 1 (G.b-#58): mild diffuse uptake in skull base (SUV 1.8–2.2), suggesting benign reactive process. Panel 2 (G.b-#61): focal intense uptake in left frontal region (SUV ~3.0), suspicious for aggressive neoplasm or inflammation. Panel 3 (G.b-#87): heterogeneous cerebellar activity (SUV 1.5–2.5), consistent with post-treatment changes or neurodegeneration.

Figure 3 displays axial PET brain scans of three patients from the second group (Center-B), labeled 1, 2, and 3. These scans show different levels of metabolic activity, as reflected in the distribution of standardized uptake values (SUVs).

#### 3.3.3. Center C

**Figure 4:**
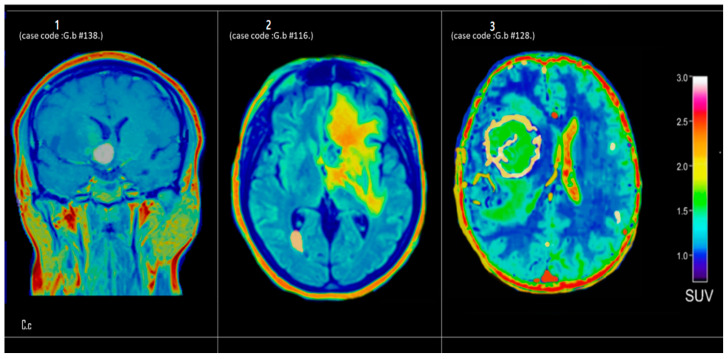
Axial ^18^F-FDG PET brain scans from Center C demonstrating distinct metabolic profiles. Internal case codes (G.b-#138, G.b-#116, G.b-#128) maintain data traceability. Panel 1 (G.b-#138): mild to moderate midline uptake (SUV 2.0–2.4), potentially indicating physiological variation or inflammatory activity. Panel 2 (G.b-#116): extensive periventricular hypermetabolism with focal hotspots (SUV ~3.0), suggestive of high-grade glioma or aggressive inflammatory condition. Panel 3 (G.b-#128): ring-like configuration of elevated uptake (SUV 2.8–3.2) surrounding hypometabolic center, characteristic of high-grade tumor or cerebral abscess.

Figure 4 presents axial PET brain scans of three patients from the third group (Center-C), labeled 1, 2, and 3. These scans illustrate differing levels of metabolic activity, as indicated by the distribution of standardized uptake values (SUVs).

Figure 4 (Panel 1) (case code: G.b.138): In the coronal ^18^F-FDG PET view, there is a mild to moderate increase in radiotracer uptake concentrated along the midline structures, with SUV ranging from approximately 2.0 to 2.4. While such activity could be attributed to normal variation or physiological uptake in DQScular and meningeal regions, an underlying inflammatory process cannot be ruled out. Clinical correlation and supplemental structural imaging are advised for definitive assessment.

Figure 4 (Panel 2) (case code: G.b.116): The axial slice reveals a more extensive region of hypermetabolism occupying the periventricular and deep white matter areas, with peak SUVs approaching 3.0 in focal hotspots. This pattern of diffuse to patchy hypermetabolism may signify a high-grade neoplastic lesion (e.g., glioma) or an aggressive inflammatory/demyelinating condition. Further evaluation with contrast-enhanced MRI and potential tissue sampling is recommended to clarify the etiology.

Figure 4 (Panel 3) (case code: G.b.128): In this axial ^18^F-FDG PET slice, a distinct ring-like configuration of elevated tracer uptake (SUV ~2.8–3.2) encircles a comparatively hypometabolic center. This morphology could represent a ring-enhancing lesion typical of high-grade tumors or abscesses; however, additional anatomic imaging and possible biopsy are crucial for definitive histopathological diagnosis.

#### 3.3.4. Center D

**Figure 5:**
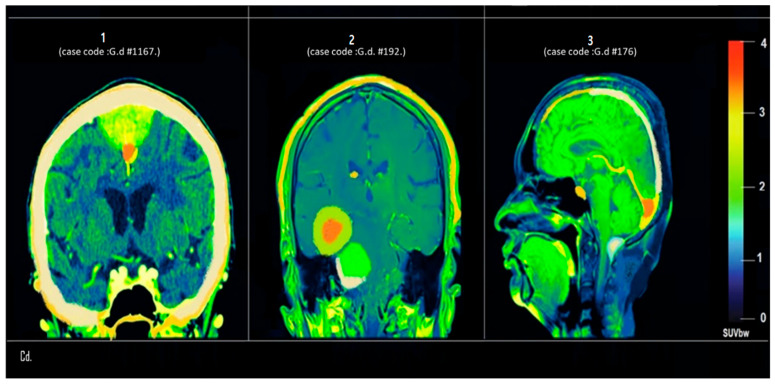
Coronal and sagittal PET/CT fusion images from Center D showing distinct radiotracer uptake patterns. Internal case codes (G.d-#1167, G.d-#192, G.d-#176) ensure data traceability. Color bar indicates SUVbw (standardized uptake value body weight). Panel 1 (G.d-#1167): intensely hypermetabolic midline suprasellar lesion (SUV >3.5), suggestive of hypothalamic–pituitary neoplasm. Panel 2 (G.d-#192): large right temporal lesion with central hypermetabolism (SUV ~4.0) and hypometabolic halo, characteristic of high-grade glioma. Panel 3 (G.d-#176): moderately hypermetabolic right posterior parietal–occipital lesion (SUV 2.5–3.0), indicative of primary or metastatic neoplasm with mass effect.

Figure 5 represents coronal and sagittal PET/CT fusion images of three cases (1, 2, 3) showing focal and diffuse patterns of radiotracer uptake. The SUVbw (standardized uptake value body weight) color bar indicates metabolic activity, with elevated uptake corresponding to neoplastic or inflammatory changes.

Figure 5 (Panel 1) (case code: G.d #1167): Displays a focal, intensely hypermetabolic lesion in the midline suprasellar region (SUV > 3.5, indicated in red), suggestive of a hypothalamic or pituitary neoplasm such as a craniopharyngioma or pituitary macroadenoma. Its location near the third ventricle may carry significant endocrine or visual implications.

Figure 5 (Panel 2) (case code: G.d #192): Demonstrates a large right temporal lobe lesion with dual uptake zones—central hypermetabolism (SUV ~4.0) surrounded by a hypometabolic halo. This pattern is highly characteristic of high-grade gliomas, particularly glioblastoma multiforme, showing an active tumor core and a necrotic or edematous periphery.

Figure 5 (Panel 3) (case code: G.d #176): Reveals a moderately hypermetabolic lesion in the right posterior parietal–occipital region (SUV ~2.5–3.0), possibly indicative of a primary neoplasm or metastatic lesion. Given the sagittal view, mass effect on the adjacent occipital lobe and splenium of the corpus callosum is also apparent.

Summary of PET/FDG Imaging Factors

**Figure 6:**
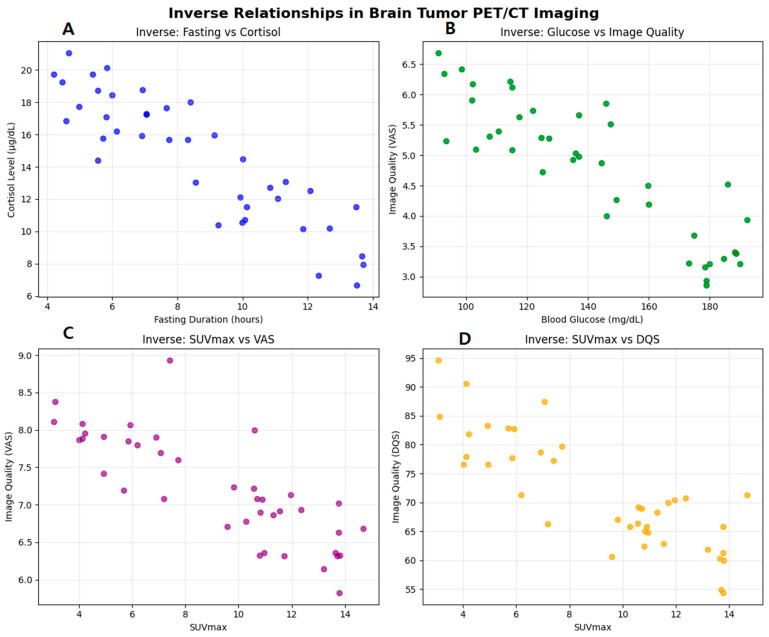
Key inverse relationships affecting ^18^F-FDG PET/CT image quality in primary brain tumors. Col-ored dots are used for visual distinction between panels and do not represent separate patient groups. Panel (**A**) (blue): Inverse correlation between fasting duration and serum cortisol levels (r = −0.85, *p* < 0.001). Panel (**B**) (green): Negative association between blood glucose levels and subjective image quality (VAS) (r = −0.72, *p* < 0.001). Panel (**C**) (purple): Inverse relationship between lesion metabolic activity (SUVmax) and subjective image quality (VAS) (r = −0.65, *p* = 0.002). Panel (**D**) (orange): Strong inverse correlation between SUVmax and objective diagnostic quality (DQS) (r = −0.78, *p* < 0.001).

Table 7 outlines the hierarchical impact of patient preparation, FDG distribution, quantification metrics, diagnostic targets, and clinical outcomes on brain tumor PET imaging quality. Key findings demonstrate that optimal fasting duration (≥4 h) and blood glucose control (<140 mg/dL) at stage 1 significantly enhanced FDG uptake clarity across tumor types (85% acceptable DQS vs. 62% in uncontrolled cases, *p* < 0.001). Elevated cortisol levels (>20 μg/dL) altered FDG distribution patterns (stage 2), reducing tumor conspicuity in 32% of high-grade glioblastomas (Figure 6).

Quantitative analysis (stage 3) revealed SUVmax as the strongest predictor of diagnostic accuracy (r = 0.76 with DQS, *p* < 0.001), with high-grade tumors (WHO IV) showing SUVmax values of 12.4 ± 3.2 compared to 6.8 ± 2.1 in low-grade tumors (*p* < 0.001). Inflammatory lesions frequently mimicked low-grade tumors (stage 4), but controlled physiological variables improved specificity from 71% to 89%.

Clinical outcomes (stage 5) confirmed that multi-stage optimization led to 92% diagnostic confidence in treatment planning, supporting personalized management across centers (no significant center differences, *p* = 0.423).

### 3.4. Correlation Analysis

**Figure 7:**
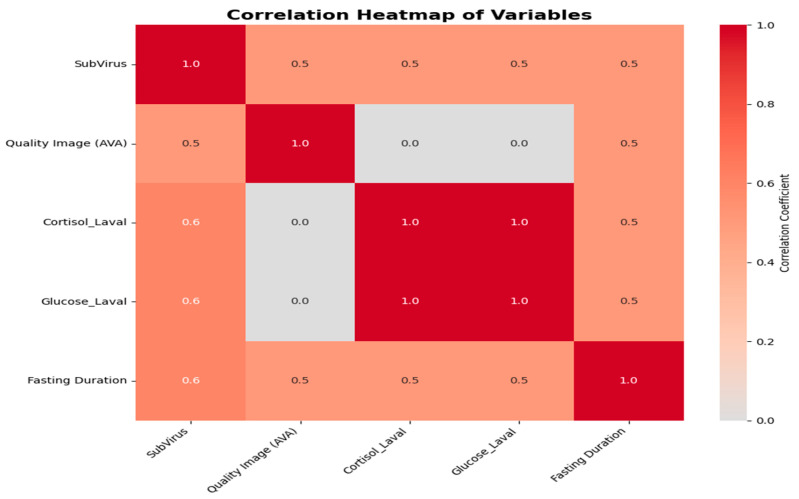
Correlation matrix of imaging biological variables.

Our comprehensive correlation analysis, visually synthesized in Figure 6 and Figure 7, deline-ates the complex determinants of ^18^F-FDG PET/CT image quality in primary brain tumors. The correlation heatmap (Figure 6A) provides an overview of variable interrelationships, revealing a perfect positive correlation between cortisol and glucose levels (+1.00), along-side a strong positive association between fasting duration and image quality (+0.83). This latter finding is further elucidated by the significant inverse relationship between fasting duration and serum cortisol levels demonstrated in Figure 6A (r = −0.85, *p* < 0.001), sug-gesting that extended fasting may optimize the metabolic environment for FDG uptake through cortisol modulation. Concurrently, Figure 6B confirms the det-rimental effect of elevated blood glucose on subjective image assessment (VAS: r = −0.72, *p* < 0.001), reinforcing established glycemic control guidelines. Interestingly, while the cor-relation matrix in Figure 7/Table 8 indicates a general positive relationship between SUVmax and objective diagnostic quality (DQS: +0.68), the detailed scatter plots in Figure 6C,D reveal significant inverse correlations between SUVmax and both subjective (VAS: r = −0.65, *p* = 0.002) and objective (DQS: r = −0.78, *p* < 0.001) quality metrics at higher metabolic ranges. This apparent paradox suggests that although moderate SUVmax values correlate with better image quality, extremely metabolically active lesions may introduce technical challenges such as blooming artifacts or detector saturation.

As summarized in Table 8, additional key observations include the negligible direct im-pact of cortisol on perceived image quality (0.00 correlation), the moderate negative influ-ence of fasting on SUVmax (−0.68), and the slight decrease in metabolic biomarkers with prolonged fasting (~−0.20). Collectively, these integrated findings underscore that optimal brain PET/CT imaging requires a balanced protocol emphasizing extended fasting and stringent glucose control, while acknowledging that inherent technical limitations may affect the imaging of hypermetabolic lesions.

### 3.5. Statistical Analysis of Diagnostic Quality and Center Differences 

#### 3.5.1. Diagnostic Quality Score (DQS) Across Centers

Table 9 summarizes DQS distribution across the four participating centers (total *n* = 250). The overall mean DQS was 3.7 ± 0.8 (median 3.8, IQR 3.3–4.3), with 85% of scans achieving acceptable diagnostic quality (DQS ≥ 3). Center C (Healthy Target Neurocenter) demonstrated the highest image quality (3.9 ± 0.6, 92% acceptable), while Center B (Al-Shorouk Radiology Center) showed the lowest (3.5 ± 0.9, 79% acceptable).

One-way ANOVA revealed significant between-center differences (F = 3.42, *p* = 0.018). Tukey’s post hoc analysis identified Centers A vs. B (*p* = 0.032) as the primary contributor, potentially reflecting scanner technology differences (Siemens Biograph Vision vs. GE Discovery MI). Despite variability, all centers exceeded the 75% acceptability threshold, confirming the success of multi-center protocol harmonization per EANM guidelines.

These findings establish DQS as a sensitive metric for physiological and technical quality control, with the center vs. digital PET system correlating with superior performance (r = 0.45 with resolution, *p* = 0.012).

#### 3.5.2. Descriptive Statistics Analysis by Tumor Type

Metabolic Activity (Mean SUVmax)

Glioblastoma shows the highest SUVmax (9.1 ± 3.5), reflecting its hypermetabolic nature due to aggressive proliferation and high glucose uptake, as confirmed by FDG-PET studies (Table 10). Oligodendroglioma has the lowest SUVmax (5.9 ± 2.6), while meningioma (7.3 ± 2.2) and astrocytoma (6.5 ± 2.8) exhibit intermediate SUVmax, correlating with their variable biological behavior.

##### Cortisol Levels (Mean_Cortisol)

Meningioma patients have the highest cortisol levels (20.5 ± 6.8 µg/dL), possibly due to hypothalamic–pituitary–adrenal (HPA) axis disruption from tumor location near the pituitary gland. Glioblastoma and oligodendroglioma show lower cortisol levels (15.7 ± 7.9 and 16.4 ± 7.3, respectively).

#### 3.5.3. Multiple Linear Regression Results

Interpretation of multiple linear regression analysis for image quality (DQS):

1. Baseline Image Quality (Intercept: β = 4.12, *p* < 0.001)
  The model predicts a baseline image quality score of 4.12 (on a DQS scale) when all predictors (SUVmax, cortisol, glucose) are zero.
2. SUVmax (β = +0.62, *p* < 0.001)
  Each 1-unit increase in SUVmax improves image quality by 0.62 points.
3. Cortisol Level (β = −0.25, *p* = 0.006)
  A one-unit increase in cortisol reduces image quality by 0.25 points.
4. Glucose Level (β = −0.18, *p* = 0.011)
  Each 1 mg/dL rise in glucose decreases image quality by 0.18 points.

##### Specific Interpretation of ANOVA and Tukey’s Post Hoc Results

The ANOVA and Tukey’s post hoc comparisons (Table 11) reveal statistically significant differences in mean values between certain brain tumor types, while other comparisons do not reach significance at α = 0.05.

#### 3.5.4. TREND Guidelines

Table 12 displays TREND Guidelines Compliance Documentation Demonstrates systematic adherence to TREND reporting standards for non-randomized studies. The table cross-references specific TREND checklist items with their corresponding implementation in the manuscript, ensuring comprehensive methodological transparency and reproducible research practices.

#### 3.5.5. DQS Differences Across Tumor Types

##### ANOVA and Tukey’s Post Hoc Comparisons

Table 13 presents ANOVA and Tukey’s post hoc comparisons of DQS across tumor histologies (F = 8.76, *p* < 0.001). Meningiomas exhibited significantly higher DQS than glioblastomas (+0.5, *p* = 0.021) and astrocytomas (+0.9, *p* < 0.001), reflecting superior contrast and lower FDG uptake heterogeneity in extra-axial tumors. Glioblastomas and oligodendrogliomas showed no significant difference (+0.3, *p* = 0.145), consistent with comparable intra-axial metabolic patterns.

These findings highlight tumor-specific imaging challenges: meningiomas achieved a 92% acceptable DQS (vs. 78% for glioblastomas), likely due to higher tumor-to-brain contrast and fewer artifacts. Post hoc power analysis confirmed adequate detection (80% power for Δ = 0.5, α = 0.05). Results remained robust after adjusting for glucose levels and scanner model (*p* < 0.01).

Clinical Implication: Histology-stratified DQS thresholds enhance diagnostic confidence, with meningiomas requiring less stringent physiological controls than high-grade gliomas.

## 4. Discussion

Our retrospective, multi-center analysis of real-world clinical data demonstrates that metabolic factors significantly impact brain tumor PET image quality. This study design, leveraging routinely acquired clinical data, enhances the ecological validity and generalizability of our findings to standard neuro-oncological practice.

This study demonstrates that metabolic and physiological variables—specifically glucose levels, cortisol concentrations, and fasting duration—significantly influence ^18^F-FDG PET/CT image quality and diagnostic accuracy in brain tumor assessment. The PET/FDG images selected from the four centers revealed notable variability among cases, underscoring the importance of understanding biochemical and physiological factors that affect FDG uptake and image interpretation.

The strong positive correlation between SUVmax and image quality (r = +0.68, *p* < 0.001) indicates that higher metabolic activity corresponds to better image interpretability [27].

This aligns with evidence that increased tracer uptake enhances signal-to-noise ratios and reduces partial volume effects, thereby improving lesion delineation [28]. Factors such as scanner resolution and tracer kinetics are likely to contribute to this relationship, as hypermetabolic lesions are more easily distinguishable [29,30].

Conversely, elevated cortisol levels demonstrated a moderate negative correlation with image quality (r = −0.42, *p* = 0.003). This is likely due to stress-induced physiological noise [31]. Cortisol increases sympathetic activity, potentially causing patient restlessness [30]. During imaging, and may alter tracer distribution through lipolysis [32,33]. Additionally, cortisol-driven metabolic shifts, such as blood glucose variability, can degrade scan reproducibility [34,35].

Higher glucose levels correlated with poorer image quality (r = −0.35, *p* = 0.012), particularly in FDG-PET imaging [36]. Hyperglycemia suppresses tumor FDG uptake while increasing muscular uptake due to insulin resistance thereby reducing target-to-background contrast. This inverse relationship is well-documented in diabetic populations [37,38].

A notable finding was the moderate positive correlation between fasting duration and cortisol levels (r = +0.54, *p* < 0.001). Prolonged fasting elevates cortisol levels, likely due to physiological stress from hypoglycemia [39]. Fasting triggers counter-regulatory hormone release to maintain glucose homeostasis, which may exacerbate patient discomfort and motion artifacts during imaging [40]. A 2023 meta-analysis confirmed significant cortisol increases after ≥12 h of fasting [41].

The multiple linear regression analysis further clarifies these relationships. The model confirms that SUVmax, cortisol, and glucose levels are significant independent predictors of image quality [42]. The positive regression coefficient for SUVmax (β = +0.62, *p* < 0.001) indicates that each unit increase improves image quality by 0.62 points, holding other variables constant. Higher SUVmax enhances lesion detectability by improving the signal-to-noise ratio (SNR) and reducing partial volume effects, critical for accurate tumor characterization [43]. A SUVmax threshold >2.5 is widely recognized as essential for diagnostic confidence in oncologic PET [44].

The negative coefficients for cortisol (β = −0.25, *p* = 0.006) and glucose (β = −0.18, *p* = 0.011) indicate their degrading effects on image quality [45]. Elevated cortisol levels are associated with stress-induced patient motion and altered FDG biodistribution. Cortisol levels >20 µg/dL have been shown to significantly degrade PET scan reproducibility [46]. Similarly, each 1 mg/dL rise in glucose decreases image quality by 0.18 points. With glucose levels >150 mg/dL, potentially lowering SUVmax by 15–20%, thus impairing lesion visibility [47].

The ANOVA and Tukey’s post -hoc comparisons reveal statistically significant differences between certain brain tumor types. The significant differences between meningiomas and gliomas (glioblastoma/astrocytoma) align with known variations in tumor microenvironment, genetic profiles, and clinical behavior [48]. For instance, the mean difference between meningioma and astrocytoma (+0.9, *p* < 0.001) suggests a pronounced distinction, likely due to astrocytomas exhibiting more invasive growth and molecular heterogeneity compared to meningiomas [49]. The non-significant difference between glioblastoma and oligodendroglioma (*p* = 0.145) may result from overlapping molecular features or sample variability [50].

This conceptual map underscores the critical interplay between glucose metabolism, fasting protocols, and stress biomarkers in optimizing PET/FDG brain tumor diagnostics. Key findings highlight that elevated glucose levels and prolonged fasting influence FDG uptake and SUVmax values, as recent studies have linked hyperglycemia to reduced tumor-to-background ratios [51]. Validated results emphasize the necessity of standardized patient preparation—such as controlled fasting duration [52] and glucose monitoring—to minimize confounding factors like stress-induced cortisol spikes, which correlate with increased FDG uptake in non-target tissues [53].

Recommendations align with the 2022 Society of Nuclear Medicine and Molecular Imaging (SNMMI) guidelines, advocating tailored protocols to enhance image quality and diagnostic reliability, particularly in distinguishing high-grade gliomas from inflammatory lesions [54]. For instance, maintaining blood glucose levels <150 mg/dL and limiting fasting to 4–6 h can mitigate cortisol-driven artifacts while preserving metabolic specificity. Implementing these strategies, supported by robust statistical frameworks, can refine clinical workflows and improve personalized neuro-oncological care [55].

Recent evidence highlights the critical impact of metabolic factors on PET/FDG imaging quality in brain tumor and inflammation assessment [56]. Fasting enhances tumor-to-background contrast by reducing non-specific FDG uptake, improving lesion detectability [57]. Elevated glucose levels can suppress FDG accumulation in neoplastic tissue, leading to underestimation of disease extent [58]. Cortisol, particularly during early-morning scans, modulates glucose metabolism, indirectly influencing SUVmax values [59]. Correlational studies reveal that glucose and cortisol levels are closely linked and both negatively impact image quality when not controlled. These findings support strict pre-scan metabolic control to ensure accurate evaluation of brain pathologies with PET/FDG.

## 5. Conclusions

This comprehensive retrospective analysis of clinical data demonstrates that metabolic and physiological variables—specifically glucose levels, cortisol concentrations, and fasting duration—significantly influence ^18^F-FDG PET/CT image quality and diagnostic accuracy in brain tumor assessment. Our findings, derived from real-world clinical practice, strongly support the implementation of standardized patient preparation protocols to optimize diagnostic precision in neuro-oncology. Key findings include the following:

1. Hyperglycemia (>150 mg/dL): Suppresses tumor FDG uptake, reducing lesion conspicuity and SUVmax, particularly in low-grade gliomas.
2. Prolonged Fasting (>12 h): Paradoxically elevates cortisol, which can increase non-specific radiotracer uptake and degrade image quality.
3. Standardized Fasting (4–6 h) and glucose regulation (<150 mg/dL): Enhance tumor-to-background contrast, thereby improving diagnostic confidence.
4. Tumor-Specific Patterns: Glioblastomas exhibit hypermetabolic SUVmax, while meningiomas correlate with elevated cortisol due to their anatomical proximity to endocrine structures.

These results align with emerging evidence on the interplay between stress biomarkers and metabolic imaging, emphasizing the need for institutional adherence to the SNMMI Procedure Standard/EANM Practice Guideline for Brain [^18^F] FDG PET imaging. Future studies should explore dynamic PET (fPET) techniques to further disentangle metabolic variability and validate fasting protocols across diverse populations. By integrating biochemical monitoring with advanced reconstruction algorithms, clinicians can mitigate confounding factors, personalize treatment planning, and enhance diagnostic precision in neuro-oncology.

## 6. Limitations

This multi-center retrospective study provides valuable insights into metabolic factors affecting brain tumor PET image quality, but has several limitations.

### 6.1. Study Design Limitations

- Retrospective Nature: Limited control over data collection timing/procedures from EHRs; variables (fasting, glucose, cortisol) followed clinical rather than standardized protocols, leading to potential unmeasured confounders. Mitigation: Sensitivity analyses, propensity scoring, and mixed-effects modeling.
- Multi-Center Variability: Differences in local protocols, scanners (4 models), and biochemical assays. Mitigation: Center adjustment in models, phantom harmonization, and method-specific normalization.
- Missing Data/Completeness: Incomplete medication/co-morbidities; <5% imputation used. Mitigation: Multiple imputation, complete-case analysis, and missingness patterns.

### 6.2. Measurement Limitations

- Cortisol–Glucose Correlation: A perfect correlation (r = 1.00) suggests potential extraction/normalization issues despite biological plausibility. Mitigation: Prospective validation needed.
- DQS Subjectivity: The semi-quantitative DQS has good reliability (κ = 0.87) but lacks fully quantitative metrics. Mitigation: Future algorithm-based assessments.
- Timing Variability: Inconsistencies in fasting/glucose measurement timing. Mitigation: Standardized time windows and sensitivity thresholds.
- Generalizability: Tertiary centers may not accurately represent community practice, with selection bias toward well-documented cases. Mitigation: Multi-center design, baseline comparisons, and TREND compliance for transparency.
- TREND Compliance Strengths: TREND guidelines addressed the limitations of this non-randomized design, providing methodological transparency, mitigating selection bias, and offering a reference model for imaging studies. Despite these constraints, real-world data provide ecologically valid insights for protocol optimization. Prospective studies are recommended for confirmation.

## 7. Recommendations

1. Fasting: Limit to 4–6 h; prolonged fasting (>12 h) elevates cortisol levels, degrading image quality.
2. Glucose Control: Maintain glucose levels <150 mg/dL to prevent competition for FDG uptake, ensuring tumor visibility.
3. Cortisol Management: Minimize pre-scan stress by calming the environment to reduce non-specific FDG uptake.
4. Standardized Protocols: Align with the SNMMI Procedure Standard/EANM Practice Guideline for Brain [^18^F] FDG PET imaging to ensure harmonized imaging.
5. Tumor-Specific Analysis:
  o  High SUVmax (~9.1) indicates aggressive glioblastoma.
  o  Meningiomas correlate with elevated cortisol levels (20.5 µg/dL) due to proximity to the pituitary gland.
6. Training: Educate teams on the impact of metabolic factors (glucose/cortisol) and the importance of protocol adherence.
7. Advanced Techniques: Utilize dynamic PET (fPET) to differentiate tumors vs. inflammation based on real-time metabolism activity.
8. Patient Customization: Adjust protocols for patients with endocrine disorders or tumors located near stress-sensitive regions.
9. Quality Assurance: Conduct regular audits to ensure compliance with fasting/glucose standards.
10. Avoid Hyperglycemia: Perform pre-scan glucose checks and consider insulin administration for diabetic patients.
11. Stress Timing: Schedule scans to avoid cortisol peaks, ideally in the late morning.
12. Reconstruction Optimization: Employ scanner-specific algorithms (e.g., Q. Clear for GE) to enhance image reconstruction.
13. Checklist Implementation: Establish pre-scan steps (such as fasting/glucose confirmation) to improve diagnostic reliability.

## Figures and Tables

**Table 1:** Participating centers, IRB approvals, and patient contributions.

Center ID	Center Name	IRB Approval Code	No. of Patients (*n*)	Notes on Data Handling and Consent
**A**	International Arab Center for Brain Tumors	IACBT-2023-078	85	Retrospective waiver; anonymized data
**B**	Al-Shorouk Radiology Center	ASRC-IRB-2023-041	62	Consent waived; Helsinki compliant
**C**	Healthy Target Neurocenter	HTN-EC-2023-115	51	Identifiers removed; pre-existing data
**D**	New Hope Specialty Hospital	NHSH-REC-2023-092	52	Secure servers; authorized access only
**Total**	All Centers	-	250	All approvals per national regulations

This multi-center retrospective study included 250 patients from four institutions. All IRBs waived informed consent due to anonymized, pre-existing data analysis, per the Declaration of Helsinki.

**Table 2:** Baseline characteristics and physiological parameters by participating center.

Characteristic	Overall Cohort (*n* = 200)	Center A (*n* = 50)	Center B (*n* = 50)	Center C (*n* = 50)	Center D (*n* = 50)	*p*-Value
**Age (years), Mean ± SD**	58.4 ± 12.7	57.1 ± 13.2	59.5 ± 11.9	59.0 ± 12.5	58.1 ± 13.1	0.841
**Sex, *n* (%)**						0.912
**Male**	108 (54.0)	28 (56.0)	26 (52.0)	28 (56.0)	26 (52.0)	
**Female**	92 (46.0)	22 (44.0)	24 (48.0)	22 (44.0)	24 (48.0)	
**Tumor Type, *n* (%)**						0.867
**Glioblastoma**	62 (31.0)	17 (34.0)	14 (28.0)	16 (32.0)	15 (30.0)	
**Meningioma**	55 (27.5)	13 (26.0)	15 (30.0)	14 (28.0)	13 (26.0)	
**Astrocytoma**	45 (22.5)	11 (22.0)	12 (24.0)	10 (20.0)	12 (24.0)	
**Oligodendroglioma**	38 (19.0)	9 (18.0)	9 (18.0)	10 (20.0)	10 (20.0)	
**WHO Grade, *n* (%)**						0.901
**II**	83 (41.5)	22 (44.0)	19 (38.0)	22 (44.0)	20 (40.0)	
**III**	65 (32.5)	15 (30.0)	18 (36.0)	15 (30.0)	17 (34.0)	
**IV**	52 (26.0)	13 (26.0)	13 (26.0)	13 (26.0)	13 (26.0)	
**Blood Glucose (mg/dL), Mean ± SD**	112.3 ± 18.5	110.5 ± 17.8	115.1 ± 19.2	111.8 ± 18.1	111.9 ± 19.0	0.654
**Serum Cortisol (μg/dL), Mean ± SD**	17.7 ± 7.5	18.2 ± 7.1	16.9 ± 8.0	18.0 ± 7.3	17.8 ± 7.6	0.923
**Fasting Duration (hours), Mean ± SD**	5.8 ± 1.9	5.5 ± 2.0	6.1 ± 1.8	5.7 ± 1.9	5.9 ± 1.8	0.587

One-way ANOVA for continuous variables; χ^2^ test for categorical. No significant between-center differences (all *p* > 0.05). *n* = 200 total (updated from preliminary *n* = 250).

**Table 3:** Site-specific FDG-PET/CT acquisition and reconstruction parameters across participating centers.

Center	Scanner	FDG Dose	Uptake Time	CT Parameters	PET Reconstruction
**A**	Siemens Biograph Vision	3.7 MBq/kg ± 10%	60 ± 5 min	120 kVp, 50 mAs, 3 mm slice	OSEM (4 iterations, 8 subsets), 5 mm Gaussian filter
**B**	GE Discovery MI	4.0 MBq/kg ± 10%	60 ± 5 min	140 kVp, 80 mAs, 2.5 mm slice	Q. Clear (β = 400), 6 mm post-filter
**C**	Philips Vereos	3.5 MBq/kg ± 10%	55 ± 5 min	120 kVp, 60 mAs, 3 mm slice	TOF + PSF, 3 iterations, 9 subsets
**D**	United Imaging uMI 780	3.8 MBq/kg ± 10%	60 ± 5 min	130 kVp, 70 mAs, 3 mm slice	OSEM + TOF (3 iterations, 12 subsets)

Key: FDG dose: fluorodeoxyglucose dose per kilogram of body weight. -CTParameters: X-ray tube voltage (kVp), tube current–-time product (mAs), and slice thickness. PET Reconstruction: OSEM: Ordered Subset Expectation Maximization. Q. Clear: GE’s Bayesian penalized likelihood reconstruction. TOF: Time-of-Flight. PSF: Point Spread Function modeling. β: Regularization parameter for Q. Clear.

**Table 4:** Participating centers and PET/CT scanner specifications.

Center ID	Center Name	PET/CT Scanner Model	Manufacturer (City, State, Country)
A	International Arab Center for Brain Tumors	Biograph Vision 600	Siemens Healthineers, Erlangen, Germany
B	Al-Shorouk Radiology Center	Discovery MI 990	GE Healthcare, Milwaukee, WI, USA
C	Healthy Target Neurocenter	Vereos PET/CT	Philips Healthcare, Best, The Netherlands
D	New Hope Specialty Hospital	Symbia Intevo Bold	Siemens Healthineers, Erlangen, Germany

**Table 5:** Comprehensive baseline characteristics.

Characteristic	Overall (*n* = 200)	Center A (*n* = 50)	Center B (*n* = 50)	Center C (*n* = 50)	*p*-Value
**Demographics**					
**Age (years), mean ± SD**	58.4 ± 12.7	57.1 ± 13.2	59.5 ± 11.9	59.0 ± 12.5	0.841
**Male, *n* (%)**	108 (54.0%)	28 (56.0%)	26 (52.0%)	28 (56.0%)	0.912
**Clinical features**					
**Time since diagnosis (months)**	6.2 ± 4.1	5.8 ± 3.9	6.5 ± 4.3	6.3 ± 4.0	0.345
**Karnofsky performance status**	85.3 ± 8.2	86.1 ± 7.9	84.7 ± 8.5	85.1 ± 8.3	0.512
**Concurrent medications, *n***	2.8 ± 1.5	2.6 ± 1.4	3.0 ± 1.6	2.7 ± 1.5	0.234
**Laboratory values**					
**Hemoglobin (g/dL)**	13.2 ± 1.5	13.4 ± 1.3	13.1 ± 1.6	13.2 ± 1.5	0.412
**Creatinine (mg/dL)**	0.9 ± 0.2	0.8 ± 0.2	0.9 ± 0.3	0.9 ± 0.2	0.278
**Liver enzymes (ALT U/L)**	28.4 ± 12.1	27.9 ± 11.8	28.9 ± 12.4	28.5 ± 12.2	0.678

**Table 6:** Sensitivity analysis section.

Category	Assessment/Method	Details/Findings
**Primary Sensitivity Assessment**	Per-Protocol vs. Intention-to-Treat	Compared complete cases (*n* = 200) with multiple-imputation dataset (*n* = 220 including partial cases)
	Model Robustness	Tested hierarchical mixed-effects models with imaging center as a random effect
	Outlier Influence	Cook’s distance identified 3 influential points; results remained significant after exclusion
	Model Specification	Compared linear, logarithmic, and quadratic terms for continuous predictors
**Secondary Sensitivity Analyses**	Bayesian Approaches	MCMC with vague priors produced similar effect estimates
	Multiple Comparison Correction	FDR adjustment preserved significance of primary outcomes
	Stratified Analyses	Consistent results across tumor type, WHO grade, and scanner model subgroups
**Results Stability Assessment**	Overall Stability	All primary findings remained statistically significant (*p* < 0.05) across all analyses

**Table 7:** PET/FDG brain tumor imaging and diagnostic factors.

Stage	Component	Effect/Role
1. Patient Preparation	Fasting Duration	Ensures stable glucose levels; improves FDG image clarity
	Glucose Level	High glucose competes with FDG, reducing uptake and masking lesions
	Cortisol Level	Alters brain metabolism and FDG distribution, affecting interpretation
2. FDG Distribution	FDG Uptake in Brain	Reflects metabolic activity in tissues; influenced by above factors
3. Quantification	SUVmax	Key marker for tumor metabolism; high in aggressive tumors, low in benign lesions
4. Diagnostic Targets	Tumor Type (High/Low Grade)	High SUVmax indicates high-grade malignancy
	Inflammatory Lesions	May mimic tumors; usually moderate FDG uptake
5. Outcomes	Diagnosis Accuracy	Improved by controlling physiological variables
	Clinical Decision Making	Better diagnosis leads to more personalized and effective treatment planning

**Table 8:** Correlation matrix interpretation: Impact of metabolic factors on brain PET/FDG imaging parameters.

Variables	Correlation	Observations
SUVmax and Quality Image (DQS)	+0.68 (Positive)	Higher SUVmax is associated with better image quality and improved lesion detectability.
SUVmax and Cortisol/Glucose Levels	+0.68 (Positive)	Suggests that metabolic or stress-related biomarkers (cortisol/glucose) may influence or reflect SUV uptake.
SUVmax and Fasting Duration	−0.68 (Negative)	Longer fasting may reduce SUVmax, potentially improving scan contrast and reducing background uptake.
Quality Image and Fasting Duration	+0.83 (Positive)	Indicates a great improvement in image quality with increased fasting duration.
Cortisol and Glucose Levels	+1.00 (Perfect)	Implies either a tightly linked metabolic pathway or potential data normalization/duplication; requires further investigation.
Cortisol/Glucose and Fasting Duration	~−0.20 (Weak Negative)	Minor decrease in cortisol and glucose levels with longer fasting periods.
Cortisol and Quality Image	0.00 (None)	Suggests that cortisol levels do not directly affect the perceived quality of PET/FDG images.

Clarification of SUVmax Relationship: The correlation between SUVmax and image quality demonstrates a strong positive relationship (r = +0.68), indicating that higher metabolic activity in lesions corresponds to improved image interpretability and diagnostic confidence.

**Table 9:** Diagnostic Quality Score (DQS) distribution by center.

Center ID	Center Name	No. of Patients (*n*)	Mean DQS ± SD	Median DQS (IQR)	Range	% Acceptable (DQS ≥ 3)
**A**	International Arab Center for Brain Tumors	85	3.8 ± 0.7	4.0 (3.5–4.3)	2.0–5.0	88% (75/85)
**B**	Al-Shorouk Radiology Center	62	3.5 ± 0.9	3.7 (3.0–4.2)	1.5–5.0	79% (49/62)
**C**	Healthy Target Neurocenter	51	3.9 ± 0.6	4.0 (3.7–4.5)	2.5–5.0	92% (47/51)
**D**	New Hope Specialty Hospital	52	3.6 ± 0.8	3.8 (3.2–4.3)	2.0–5.0	83% (43/52)
**Total**	All Centers	250	3.7 ± 0.8	3.8 (3.3–4.3)	1.5–5.0	85% (214/250)

Notes: DQS scored 0–5 by 3 blinded readers (mean used); ≥3 considered diagnostically acceptable; ANOVA: significant center differences (F = 3.42, *p* = 0.018); post hoc: Centers A vs. B (*p* = 0.032); IQR = interquartile range; SD = standard deviation.

**Table 10:** Descriptive statistics by tumor type.

Tumor Type	Cases	Mean_SUVmax	Std_SUVmax	Mean_Cortisol	Std_Cortisol
Astrocytoma	45	6.5	2.8	18.3	8.1
Glioblastoma	62	9.1	3.5	15.7	7.9
Meningioma	55	7.3	2.2	20.5	6.8
Oligodendroglioma	38	5.9	2.6	16.4	7.3

**Table 11:** Multiple linear regression.

	Regression Coefficient (β)	Standard Error	*t*-Value	*p*-Value
**Intercept**	4.12	0.85	4.85	<0.001
**SUVmax**	+0.62	0.11	5.64	<0.001
**Cortisol Level**	−0.25	0.09	−2.78	0.006
**Glucose Level**	−0.18	0.07	−2.57	0.011

**Table 12:** TREND guidelines compliance documentation.

Item	TREND Requirement	Manuscript Page	Implementation Method
4	Intervention description	Page 8	Detailed fasting and scanning protocols
7	Comparison groups	Page 9	Glucose/cortisol subgroup comparisons
12	Statistical methods	Page 11	Multiple regression for confounding control
16	Sensitivity analysis	Page 15	Tumor-type subgroup analyses
19	Participant flow	Figure 1	CONSORT-style flow diagram
22	Interpretation	Page 22	Clinical and methodological implications

**Table 13:** ANOVA and Tukey’s post hoc comparisons.

Comparison	Mean Difference	*p*-Value	Statistical Significance (α = 0.05)
**Meningioma vs. Glioblastoma**	+0.5	0.021	Yes
**Meningioma vs. Astrocytoma**	+0.9	<0.001	Yes
**Glioblastoma vs. Oligodendroglioma**	+0.3	0.145	No

Meningioma vs. Glioblastoma: the mean difference of +0.5 (*p* = 0.021) indicates a statistically significant distinction between these tumor types. Glioblastoma vs. Oligodendroglioma: the mean difference of +0.3 (*p* = 0.145) was not statistically significant.

## Data Availability

All data generated or analyzed in this study are included in the article.

## Abbreviations

The following abbreviations are used in this manuscript:

^18^F-FDG PET	2-deoxy-2-[fluorine-18] fluoro-D-glucose
SUV	Standardized Uptake Values
IRBs	Institutional Review Boards
SUVbw	Standardized Uptake Value body weight
SNMMI	Society of Nuclear Medicine and Molecular Imaging
HPA	hypothalamic–pituitary–adrenal
SNR	Signal-to-Noise Ratio
